# Supplementary material for: Immobilization of Horseradish Peroxidase on Magnetite-Alginate Beads to Enable Effective Strong Binding and Enzyme Recycling during Anthraquinone Dyes’ Degradation
Source: Polymers (Basel). 2022 Jun 28;14(13):2614. doi: 10.3390/polym14132614 (PMC9269335; doi:10.3390/polym14132614)
Supplement: Supplementary file 1 [file polymers-14-02614-s001.zip › polymers-1728563-supplementary.pdf]

Immobilization of Horseradish Peroxidase on Magnetite-Alginate Beads to Enable Effective Strong Binding and Enzyme Recycling during Anthraquinone Dyes' Degradation

Marko Jonović [1], Branimir Jugović [2], Milena Žuža [3], Verica Đorđević [4], Nikola Milašinović [5], BBranko Bugarski [4] and Zorica Knežević-Jugović [3,*]

1 Institute of Chemistry, Technology and Metallurgy, University of Belgrade, Njegoševa 12, 11000 Belgrade, Serbia; marko.jonovic@ihtm.bg.ac.rs
2 Institute of Technical Science of the Serbian Academy of Sciences and Arts (SASA), Knez Mihailova 35/IV, 11000 Belgrade, Serbia; branimir.jugovic@itn.sanu.ac.rs
3 Department of Biochemical Engineering and Biotechnology, Faculty of Technology and Metallurgy, University of Belgrade, Karnegijeva 4, 11000 Belgrade, Serbia; mzuza@tmf.bg.ac.rs
4 Department of Chemical Engineering, Faculty of Technology and Metallurgy, University of Belgrade, Karnegijeva 4, 11000, Belgrade, Serbia; vmanojlovic@tmf.bg.ac.rs (V.Đ.); branko@tmf.bg.ac.rs (B.B.)
5 Department of Forensic Engineering, University of Criminal Investigation and Police Studies, Cara Dušana 196, 11080 Belgrade, Serbia; nikolla.milasinovic@kpu.edu.rs

* Correspondence: author: zknez@tmf.bg.ac.rs; Tel.: +381-113-303-776

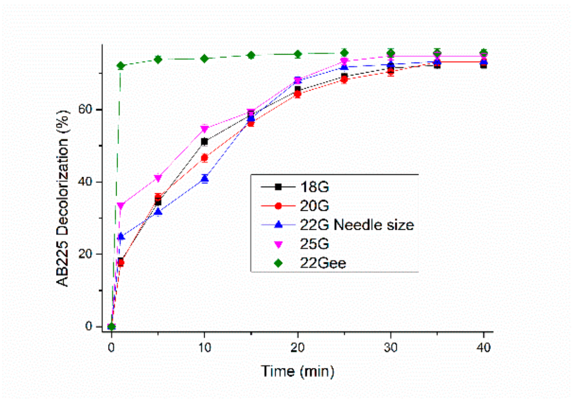

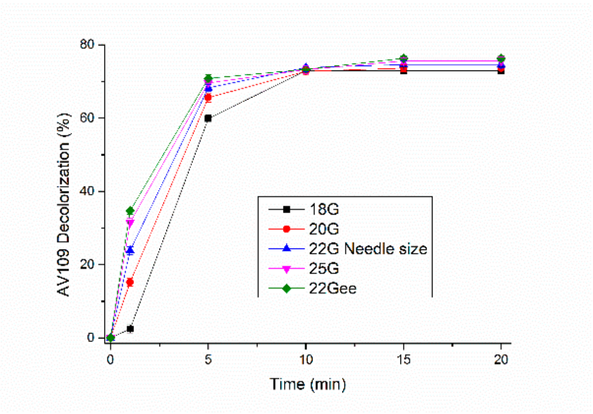

a) **b)**

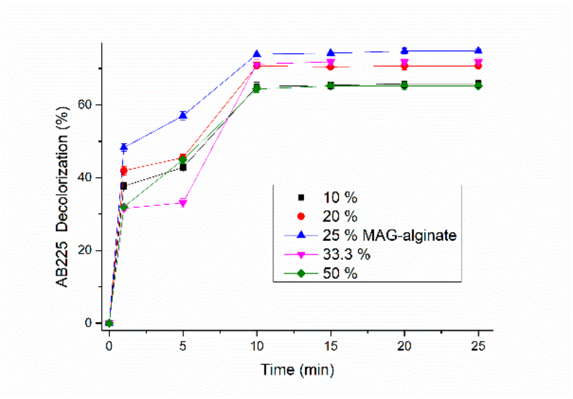

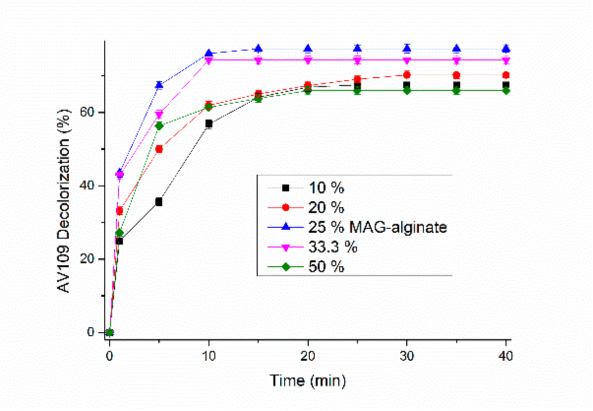

c) **d)**

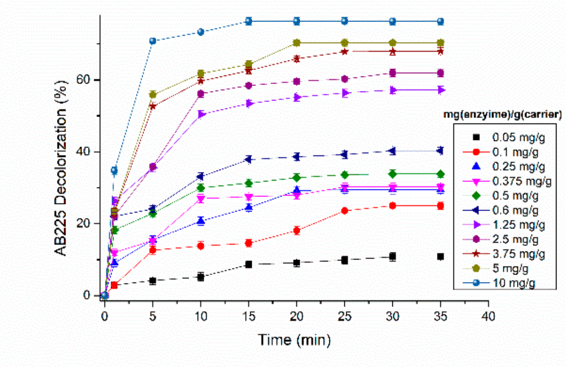
AB225 Decolorization (%)
Time (min)
mg(enzyime)/g(carrier)
0.05 mg/g
0.1 mg/g
0.25 mg/g
0.375 mg/g
0.5 mg/g
0.6 mg/g
1.25 mg/g
2.5 mg/g
3.75 mg/g
5 mg/g
10 mg/g

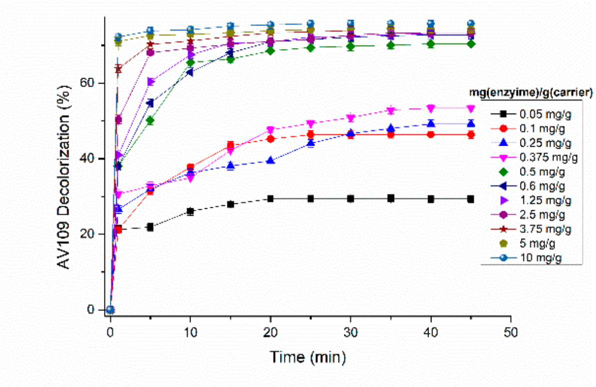
AV109 Decolorization (%)
Time (min)
mg(enzyime)/g(carrier)
0.05 mg/g
0.1 mg/g
0.25 mg/g
0.375 mg/g
0.5 mg/g
0.6 mg/g
1.25 mg/g
2.5 mg/g
3.75 mg/g
5 mg/g
10 mg/g

e) f)

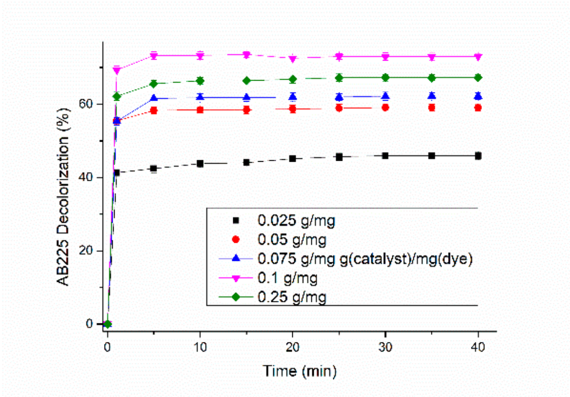
AB225 Decolorization (%)
Time (min)
0.025 g/mg
0.05 g/mg
0.075 g/mg g(catalyst)/mg(dye)
0.1 g/mg
0.25 g/mg

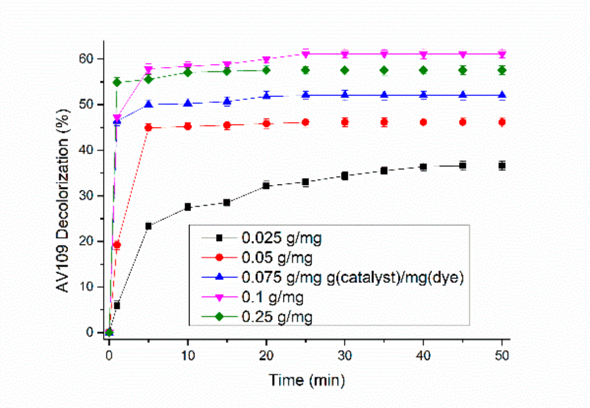
AV109 Decolorization (%)
Time (min)
0.025 g/mg
0.05 g/mg
0.075 g/mg g(catalyst)/mg(dye)
0.1 g/mg
0.25 g/mg

g) h)

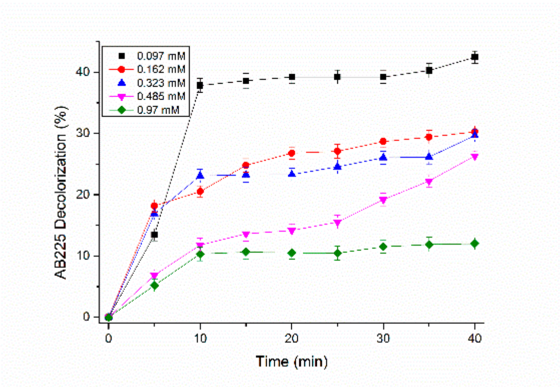
AB225 Decolorization (%)
Time (min)
0.097 mM
0.162 mM
0.323 mM
0.485 mM
0.97 mM

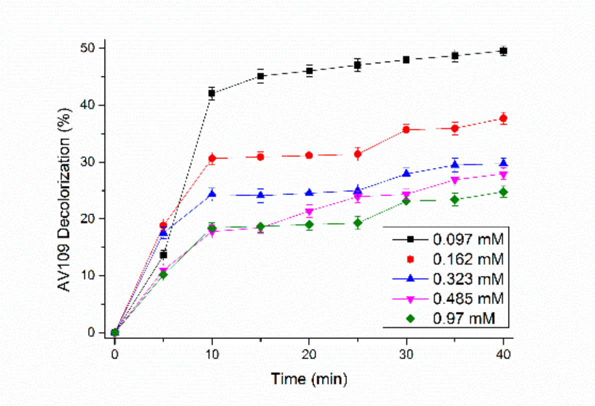
AV109 Decolorization (%)
Time (min)
0.097 mM
0.162 mM
0.323 mM
0.485 mM
0.97 mM

i) j)

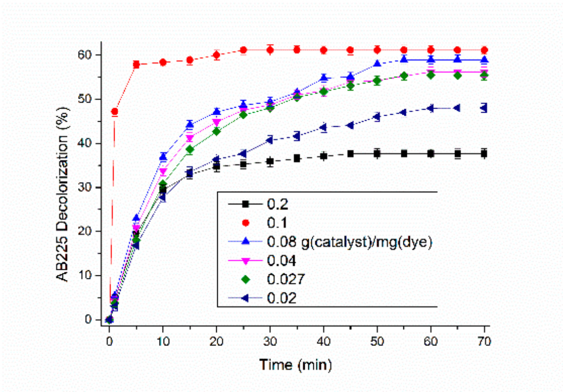
AB225 Decolorization (%)
Time (min)
0.2
0.1
0.08 g(catalyst)/mg(dye)
0.04
0.027
0.02

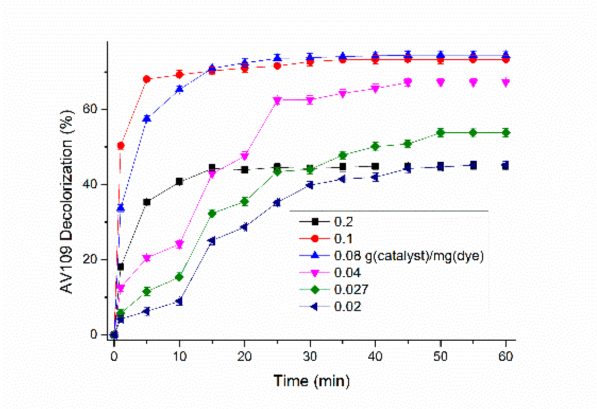
AV109 Decolorization (%)
Time (min)
0.2
0.1
0.08 g(catalyst)/mg(dye)
0.04
0.027
0.02

k) l)

Figure S1: Effects on the decolorization process: HRP-MAB size (**a**) AB225, (**b**) AV109; MAG-alginate ratio (**c**) AB225, (**d**) AV109; initial HRP concentration (**e**) AB225, (**f**) AV109; HRP-MAB mass (**g**) AB225, (**h**) AV109; initial H_2O_2 concentration (**i**) AB225, (**j**) AV109 and initial dye concentration (**k**) AB225, (**l**) AV109.

Table S1: Reaction conditions for the optimization of decolorization process of AB225 and AV109 color

Varied parameter	Time, min	MAG-alginate	$C_{peroxide}$, mM	C_{color}, g(catalyst)/mg(dye)	Beads mass, g(catalyst)/mg(dye)	C_{HRP}, mg(enzyme)/$g_{carrier}$	Nozzle size
**MAG-alginate**	AB 25 AV 40	1:10 – 1:2	0.097	0.1	0.05	2.5	$22G_{ee}$
**$C_{peroxide}$, mM**	AB 40 AV 40	1:4	0.097 – 0.97	0.1	0.025	10	*$22G_{ee}$
**C_{color}, g(catalyst)/ mg(dye)**	AB 70 AV 60	1:4	0.097	0.02 – 0.2	0.05	2.5	$22G_{ee}$
**Beads mass, g(catalyst)/mg(dye)**	AB 50 AV 40	1:4	0.097	0.1	0.025 – 0.25	2.5	$22G_{ee}$
**C_{HRP}, mg(enzyme)/ $g_{carrier}$**	AB 35 AV 45	1:4	0.097	0.1	0.05	0.05 - 10	$22G_{ee}$
**Nozzle size**	AB 40 AV 20	1:4	0.097	0.1	0.05	10	18G – $22G_{ee}$

$C_{peroxide}$ – H_2O_2 concentration; C_{color} – Dye concentration; C_{HRP} – HRP initial concentration; *ee – electrostatic extrusion
